# Nursing shortages and migration: a two-decade study of Ireland’s dependence on migrant nurses

**DOI:** 10.1016/j.hpopen.2026.100161

**Published:** 2026-01-07

**Authors:** Comfort O. Chima, Vishnu Renjith, Niamh Humphries

**Affiliations:** a School of Nursing and Midwifery, The Royal College of Surgeons in Ireland, University of Medicine and Health Sciences, Dublin, Ireland; b Manipal College of Nursing, Manipal Academy of Higher Education, India; c Graduate School of Healthcare Management, The Royal College of Surgeons in Ireland, University of Medicine and Health Sciences, Dublin, Ireland

**Keywords:** Nursing migration, Nursing recruitment, Overseas nurses, Nursing retention, Nursing training, Nursing employment

## Abstract

**Background:**

There has been global concern about nursing shortages and nurse migration, as nurses move in search of better working conditions. Ireland is one of the countries facing nursing shortages. Ireland, like many other countries, has begun to rely heavily on migrant nurses. This study examines the recent trends in the nursing workforce and possible contributing factors to Ireland’s dependence on international nurse recruitment, drawing insights from existing data and literature.

**Methodology:**

This paper integrates and analyses secondary data obtained from the Nursing and Midwifery Board of Ireland (NMBI) 2003–2022 and data from the Faculty of Nursing and Midwifery, RCSI, registration register (2015–2022). The population data of Ireland was obtained from the Central Statistics Office (CSO).

**Result:**

The data available indicates a heavy reliance on migrant nurses due to a shortage of Irish-trained nurses relative to increased demand for nurses in the period (2003–2022). More than 50% of the Nurses registered in that period were migrant nurses (EU and Non-EU).

**Conclusion:**

There is an urgent need for Ireland to increase the domestic training of nurses and midwives, as well as, a need to develop a robust nurse workforce planning system to improve retention and ensure nursing workforce self-sufficiency.

## Highlights

• Ireland continues to rely on migrant nurses and will continue to do so.
• Urgency to increase the domestic training of Irish nurses and midwives.
• A need to develop a robust nurse workforce planning system.
• A radical measure effected for self-reliance.

## 1 Background

Many countries worldwide face challenges associated with nurse shortages and nurse emigration [1,2], Ireland included. Although Ireland has successfully attracted migrant nurses in recent years, the country still faces a nursing shortage [3,4]. Several underlying multidimensional factors have contributed to the increased reliance on migrant nurses in Ireland including active international nurse recruitment campaigns [5], the growing healthcare sector and the expansion of the public acute hospital workforce (ESRI 2022), strong economic conditions and changes in workforce planning for nurses and midwives [4].

However, despite significant migrant nurse recruitment, Ireland continues to experience severe nurse shortages, affecting the country’s healthcare system [6,7,3]. Ireland’s nurse shortage is attributable to the ageing population, longer life expectancy, and increased need for nursing services in care homes, a trend predicted to continue in the future [8].

Attaining healthcare goals, healthcare coverage, and bolstering the healthcare system are dependent on a sufficient number of nurses (Alameddine et al., 2017). This is to prevent compromise on the quality of healthcare services and improve the well-being of the population in achieving health coverage [1], Alameddine et al., 2017). Insufficient nursing staff within healthcare has a detrimental impact on patient care [9].

Ireland requires an expanded nursing workforce to cater to the ageing population [10], NMBI 2024). Ageing populations are increasing among both Organisation for Economic Co-operation and Development (OECD) members and non-member countries [11,12] and with continued rapid population growth; demand for health and social care is projected to increase across all health sectors in Ireland [13].

In addition to the increased patient demand, workforce factors influence the nursing shortage. For instance, the ageing nursing workforce, nurse emigration to more attractive labour markets [5,3], inadequate implementation of retention policies, and early exits from the nursing profession also contribute to Ireland's nursing shortage [2,14,15,3].

Historically, emigration has been a defining feature of the Irish nursing profession. Since the nineteenth century, Irish nurses have migrated to destinations such as the United Kingdom, the United States, Canada, and Australia in search of higher wages and improved working conditions [16]. By the 1990s, however, Ireland was experiencing acute nursing shortages, which reversed the traditional trend and positioned the country as an importer of nursing labour [17,18]. The economic boom of the “Celtic Tiger” further intensified demand for nurses, and inward migration increased sharply. The 2004 EU enlargement facilitated the entry of nurses from Poland, Lithuania, and Romania [19], while active international recruitment campaigns targeted nurses from India and the Philippines [5].

Yet this growth proved vulnerable to economic volatility. With the collapse of the Celtic Tiger and the onset of austerity measures after 2008, public sector hiring restrictions, including a national recruitment freeze, were introduced to control health expenditure. These policies abruptly curtailed the hiring of both domestic and migrant nurses, despite persistent staffing needs [20]. As opportunities contracted, international nurses already employed in Ireland faced reduced prospects for permanent posts or professional advancement, while potential migrants increasingly redirected their pathways to other countries. Consequently, rather than stabilising the workforce, the recruitment freeze contributed to renewed outward migration, underscoring the cyclical and policy-sensitive nature of nurse mobility in Ireland.

Today, migrant nurses constitute an essential component of the Irish nursing workforce. In 2022, approximately 50 % of practicing nurses in Ireland were internationally trained [21]. Migrant nurses continue to fill critical gaps and help maintain service continuity across hospitals and community care settings [22].

In this study, the term Migrant Nurse, refers to a nurse who has crossed a national border to seek or take up employment in another country [23]. This group of nurses is often described in workforce literature as Internationally Trained Nurses (ITNs) or overseas nurses who obtained their primary nursing qualification in a country other than the one in which they are currently practising [11,12]. These internationally trained or migrant nurses form a significant component of Ireland’s nursing workforce and play a central role in addressing persistent staffing shortages.

A key mechanism supporting the integration of internationally trained nurses into the Irish workforce is the Nursing and Midwifery Board of Ireland (NMBI) registration process. The NMBI is the statutory body responsible for maintaining the professional register of nurses and midwives in Ireland [24]. Registration is open to two groups of nurses: graduates of approved Irish nursing and midwifery programmes and internationally qualified nurses whose credentials, experience, and professional competence meet NMBI standards [25].

To obtain registration, migrant nurses must complete one of two pathways. The first is the Adaptation and Assessment programme, which entails the migrant nurse completing a supervised clinical placement lasting 6 to 12 weeks in an NMBI-approved healthcare facility. During this period, applicant migrant nurses must demonstrate competency across the professional domains defined by NMBI, including clinical knowledge, ethical practice, patient safety, and professional accountability [24]. Successful completion leads to registration, whereas those who do not meet the required competencies may extend the placement or pursue alternative assessment.

The second pathway is the Aptitude Test, introduced in 2015 and administered by the Royal College of Surgeons in Ireland (RCSI). This assessment of applicant migrant nurses involves a theoretical multiple-choice examination and an Objective Structured Clinical Examination (OSCE) designed to evaluate practical skills in a simulated environment [26]. Accomplishing both components enable the nurse to join the NMBI register and commence practice in Ireland.

Given the central role of migrant nurses in sustaining the Irish healthcare system, understanding the trends and dynamics influencing their recruitment and integration is essential. Therefore, this study examines the demand and supply of migrant nurses in Ireland from 2003 to 2022, exploring the factors driving international recruitment and comparing workforce trends with population healthcare needs during this period. This analysis provides crucial insights for future nurse workforce planning and policy development.

## 2 Methods

This study utilised secondary data obtained from three key sources: the annual registration statistics published by the Nursing and Midwifery Board of Ireland (NMBI), the registration records of the Royal College of Surgeons in Ireland (RCSI) Faculty of Nursing and Midwifery, and population and workforce datasets from the Central Statistics Office (CSO). These publicly available datasets provide information on the number of nurses entering the Irish professional register between 2003 and 2022. These data allowed examination of long-term trends in international recruitment as well as demographic shifts relevant to national nursing workforce demand.

Data extraction from online sources was conducted around May – August 2023. The datasets were collated and managed using Microsoft Excel and Jupyter Notebook. Descriptive analyses, trend estimations, and graphical summaries were generated to assess patterns in nurse mobility, and associations between population dynamics, nurse density, and the supply of internationally trained nurses were explored over the study period. These analyses provide an empirical foundation for understanding workforce pressures and informing future health policy decisions.

### 2.1 Data analysis

Data were analysed using a structured, multi-step approach to ensure clarity, transparency, and reproducibility. NMBI registration data were first extracted and categorised into three groups of new entrants: Irish-trained, EU-trained, and non-EU-trained nurses. For the period 2015–2022, data on nurses entering the register through the RCSI adaptation/assessment pathway were integrated with the NMBI dataset to produce complete annual totals of newly registered nurses. Data cleaning procedures included removing duplicate entries, validating country-of-training classifications, and harmonising variables across datasets.

A longitudinal time-series dataset was then constructed for the years 2003–2022. Descriptive statistical analysis was undertaken to examine annual changes in the volume and composition of new nurse entrants. Nurse density was calculated as the number of active nurses per 1,000 population, using mid-year population estimates for each year from Ireland’s Central Statistics Organisation (CSO). This allowed comparison of workforce supply relative to population growth across the study period.

**Fig. 1:**
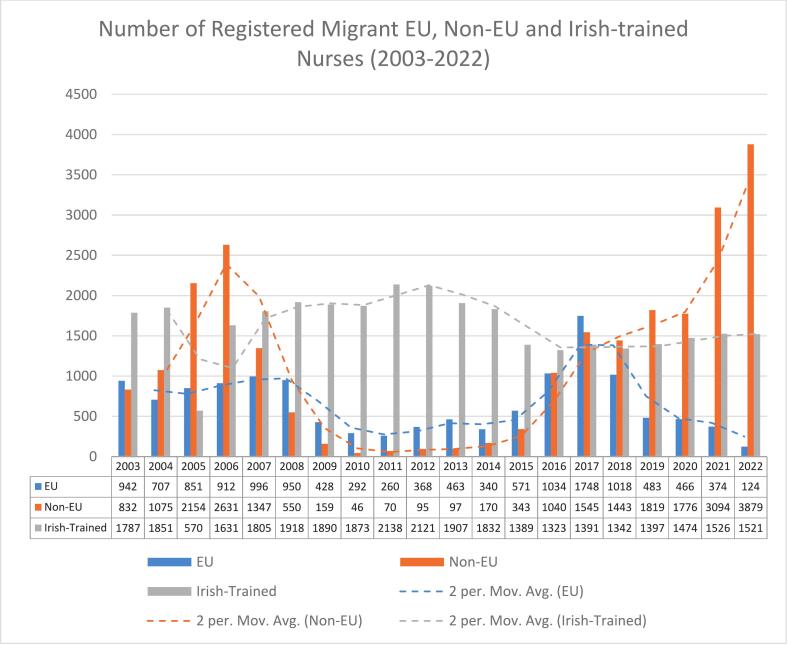
Number of Registered Migrant EU, Non-EU Migrant, and Irish-trained Nurses (2003–2022).

Source: NMBI and RCSI

**Fig. 2:**
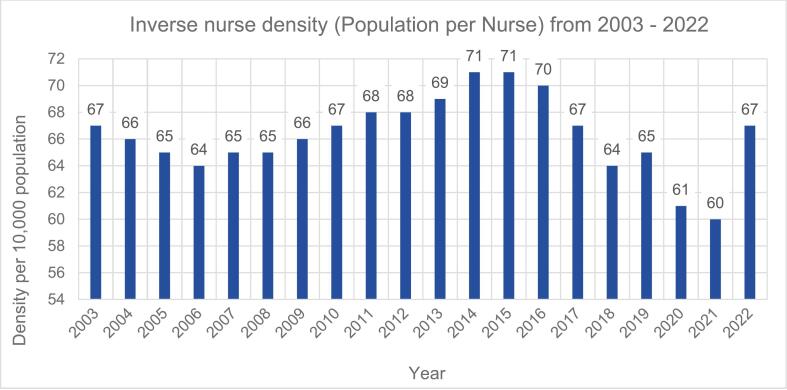
Citizens per Nurse in Ireland from 2003 to 2022.

Source: CSO and NMBI

**Fig. 3:**
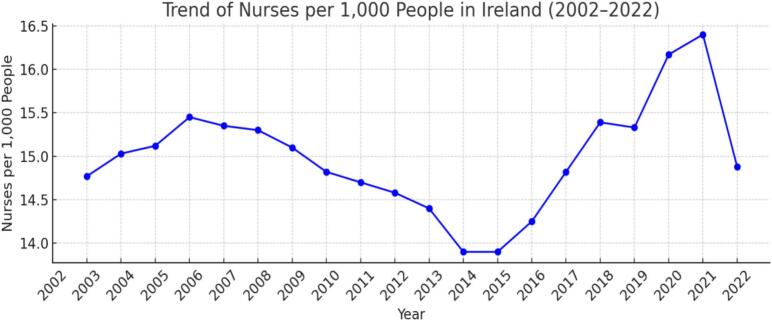
Trends of Nurses per 1000 People in Ireland (2003–2022).

Source: CSO and NMBI

**Fig. 4:**
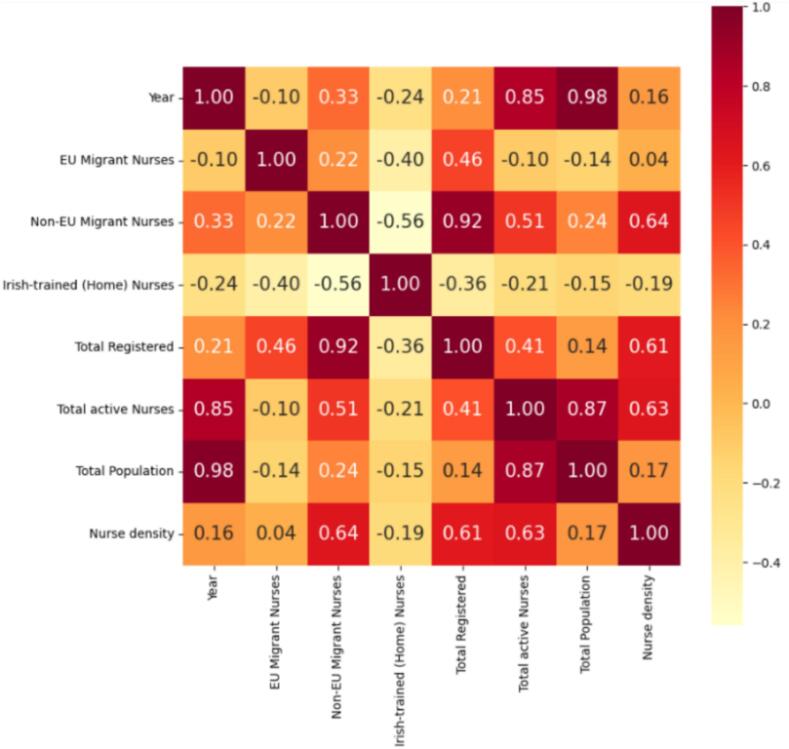
Correlation matrix showing the relationship between several factors and especially highlighting the dependence of Nurse density on Non-EU migrant Nurses.

Source: CSO and NMBI

To further explore potential relationships between workforce indicators, a correlation analysis was conducted between nurse density and the number of Irish, EU, and non-EU new entrants. A correlation matrix was generated in Jupyter Notebook to identify the relative strength and direction of associations, with particular attention to the contribution of non-EU migrant nurses to stabilising nurse-to-population ratios. All analyses and visualisations (Fig. 1, Fig. 2, Fig. 3, Fig. 4) were produced using Microsoft Excel and Jupyter Notebook.

## 3 Results

### 3.1 Profile of nurse migration to Ireland

The NMBI registration data were used to examine the flow of migrant nurses entering the Irish nursing workforce between 2003 and 2022, complemented by RCSI data from 2015 to 2022, which were incorporated into the total number of annual registrants. Professional registration data remains the most reliable measure for assessing both the contribution of migrant nurses and the overall volume of active and newly registered nurses. From 2015 onward, a clear correlation emerged between registrations completed through the RCSI pathway and the total number of new entrants to the NMBI register, reflecting the growing importance of this route for internationally trained nurses.

Each data point in Fig. 1, represents the annual number of new nurse registrants entering the Irish workforce. Across the 2003–2022 period, the number of Irish-trained, EU-trained, and non-EU-trained nurses fluctuated considerably. EU-trained nurses are those relocating from other EU member states, they experienced a significant peak in 2017, when their numbers surpassed both Irish-trained and non-EU registrants. Following this peak, EU nurse inflow declined sharply, and by 2022 this group accounted for only **7.3 %** of all new registrants.

In contrast, non-EU migrant nurses constituted the majority of new entrants in 2022, representing **61 %** of new additions to the register. Their numbers showed marked variability over the study period: an initial peak in 2006 was followed by a sustained decline, making them the smallest group of new entrants between 2008 and 2015. From 2016 onwards, however, non-EU arrivals increased substantially, with pronounced surges in 2021 and 2022.

Between 2007 and 2016, Irish-trained nurses formed the largest share of new registrants. Their entry numbers have remained relatively stable in recent years, with approximately 1,500 graduates joining the register annually. A notable exception occurred in 2005, when the transition from a three-year to a four-year nursing degree programme resulted in a temporary reduction in graduates.

Notably, 2017 marked an exceptional year in which the numbers of Irish-trained, EU, and non-EU nurses entering the Irish health system converged, with each group contributing approximately 1,300–1,800 new registrants. This rare alignment underscores the dynamic interplay of domestic training output, EU mobility, and international recruitment in shaping Ireland’s nursing workforce composition.

### 3.2 Nurses to population ratio in Ireland from 2003 to 2022

Fig. 2 above shows the inverse nurse density from 2003 to 2022. In 2003, the data indicated that there were approximately **67** people for every active nurse within the country. This means that every nurse potentially had an average of **67** people to care for. It is expected that the demand for the nursing workforce will grow with the demand for healthcare within a population. Notably, 2020 and 2021 were the only years in which the population-per-nurse ratio fell below 64.

### 3.3 Nurse density in Ireland from 2003 to 2022

To assess annual changes in workforce capacity, nurse supply was approximated using the ratio of active nurses per 1,000 population. Fig. 3 illustrates the trend in nurse density from 2003 to 2022, showing modest fluctuations between approximately **13.9** and **16.4** nurses per 1,000 people. Although both the population and the number of active nurses increased during this period, the relative stability of this ratio suggests that workforce expansion has only marginally kept pace with population growth.

A correlation analysis (Fig. 4) examining nurse density and the annual inflow of new registrants by category revealed the strongest association between nurse density and the number of non-EU entrants. This indicates that non-EU recruitment has played a decisive role in stabilising nurse-to-population ratios, with increases in non-EU registrations appearing to offset periods of lower domestic or EU-trained supply. These findings reinforce the central importance of non-EU international recruitment in maintaining workforce equilibrium within the Irish health system.

In 2003, Ireland had approximately **14.77** active nurses per 1,000 population, a figure that changed only marginally to **14.88** per 1,000 by 2022. As illustrated in Fig. 1, Fig. 3, annual fluctuations in nurse density correspond closely with changes in migrant nurse inflows, particularly those from non-EU countries, and appear to vary inversely with the supply of Irish-trained nurses. Periods of lower nurse density were typically followed by increases in migrant nurse recruitment in the subsequent year, suggesting a compensatory mechanism within the workforce system. An observable rise in nurse density occurred around 2020, peaking in 2021 during the COVID-19 pandemic, followed by a sharp decline in 2022.

The correlation matrix presented in Fig. 4 further highlights the strong association between nurse density and non-EU recruitment, demonstrating the extent to which non-EU inflows underpin Ireland’s capacity to maintain stable staffing levels. Although the total number of active nurses has increased over time, the nurse-to-population ratio has remained relatively stable, indicating that workforce expansion has only marginally kept pace with demographic growth. This relative stability reflects a health system that is dependent on international recruitment to offset domestic supply constraints.

### 3.4 Contextual factors influencing nurse supply

Several historical factors help explain these trends. The public-sector recruitment embargo between 2009 and 2013 significantly reduced permanent opportunities for newly qualified nurses and midwives, prompting many to emigrate in search of more favourable working conditions [6,18]. The subsequent Graduate Nurse Employment Scheme, introduced in 2013 to provide 1,000 posts at reduced salary rates, was widely criticised by professional bodies and had limited success in retaining early-career nurses. As domestic retention weakened, international recruitment efforts intensified to fill emerging gaps. The spike in EU nurse registrations in 2017 may also reflect Brexit-related uncertainty in the United Kingdom, which prompted some EU nurses to leave the UK’s National Health Service and seek employment in alternative jurisdictions, including Ireland (NMC, 2019; [27].

Overall, findings reveal a cyclical pattern: workforce shortages, emigration, and population pressures result in increased international recruitment, particularly from non-EU countries.

Understanding the complex interplay of domestic policy, international mobility, and demographic pressures is essential for identifying sustainable solutions to Ireland’s nursing shortages. These findings underscore the need for deeper investigation into the structural drivers of workforce instability and the development of longer-term strategies to strengthen domestic training, enhance retention, and reduce reliance on international recruitment.

## 4 Discussion

### 4.1 Profile of nurse migration to Ireland

Although nurse shortages are a global challenge [28], according to Organisation for Economic Co-operation and Development (OECD) data in 2021, Ireland is more heavily reliant on internationally trained nurses than any other OECD country [14,29].

This study examined the trend of migrant nurses joining the Irish nursing register from 2003 to 2022, the number of migrant nurses surpasses the number of Irish-trained nurses joining the nursing register. Fig. 1 highlights a concerning trend, that Ireland is not adequately training nurses to meet the growing demand of the healthcare system and population.

The 2010 World Health Organisation (WHO) global code on the international recruitment of health personnel, highlighted the need for every country to achieve self-sufficiency in the healthcare workforce. This is echoed by the International Council of Nurses (ICN) 2019 report, which emphasised the importance of each country training and retaining more of its own nurses and implementing ethical recruitment practices while employing nurses from abroad [30]. This includes funding the development of more nursing programs and providing ongoing professional development opportunities [23].

Comparing Ireland with Norway, another high-income country with a similar population, it illustrates the scale of Ireland’s training gap. Norway has substantially increased nursing student places since 2010, reaching 5,100 first-year places in 2022, while Ireland increased its intake only modestly from 1,570 in 2014 to 2,032 in 2021 [14]. Without significant investment in nurse education capacity, Ireland will remain dependent on recruitment from abroad, raising important considerations for long-term workforce sustainability and ethical recruitment practices.

### 4.2 Ongoing demand for nursing and Midwifery services in Ireland

The demand for nursing and midwifery services in Ireland will continue to rise alongside overall demand for healthcare and population growth [13]. Since 2003, life expectancy in Ireland has increased from 75 to 85 years [21]. Although a positive trend, increased life expectancy will also necessitate additional care provisions for an ageing population, with ongoing support for survivors of major illnesses, and a need to address the social care needs of the frail elderly population [8].

In 2017, the “Sláintecare programme of reforms was initiated to reform the Irish healthcare system, aiming to facilitate and ensure timely access to high-quality care. This 10-year strategy envisages a healthier Ireland, with improved health and well-being for all, with the delivery of required care in the correct health settings as appropriate [32]. Achieving these reforms depends on a sufficiently staffed nursing workforce.

The statistics presented in Fig. 2 reveal inconsistencies in the number of nurses available to meet the healthcare needs of a growing and ageing population. An important component of health workforce planning is ensuring that the number of available nurses meets population demand [33]. A failure to achieve this balance results in shortages of nurses and a reliance on migrant nurse recruitment as the solution of choice [34]. The ongoing demand and continuous need for migrant nurses within the Irish workforce from 2003 to 2022, as shown in Fig. 1, indicates nursing shortages within the health services.

Buchan & Aiken [35], posit nursing shortages as an imbalance between the requirements for nursing skills and the actual availability of nurses who are delivering safe and effective care. Meaning not necessarily a shortage of individuals with nursing qualifications, but rather a shortage of qualified nurses willing to work for the terms and conditions on offer [35]. This distinction is critical for understanding both recruitment and retention dynamics.

Literature reveals that Ireland has a relatively high number of nurses per capital (4th highest) compared to other OECD countries [14]. However, Ireland also has the highest dependence on migrant nurses. There is an urgent need for Ireland to increase the domestic training of nurses and midwives to meet projected increases in healthcare demand (ESRI 2022), this would help in addressing workforce challenges, and reduce Ireland’s reliance on migrant nurses recruitment [].

### 4.3 Impact of Covid-19 pandemic: lessons for the future workforce resilience

The dataset presented in Fig. 2, revealed a noticeable increase in the ratio of nurses per 10,000 population around 2020, reaching a peak in 2021. However, this ratio decreased in 2022, aligning closer to the levels seen in the mid-2010 s. This fluctuation reflects emergency recruitment measures and the heightened volatility of the nursing workforce during the pandemic.

The COVID-19 pandemic has revealed the critical importance of health workers for the effective delivery of health services and for an effective pandemic response [36,37,38]. The pandemic disrupted public health, the medical care system, and the global economy, shaping the work future [39]. It also, radically altered the work environment and dynamics within the health workforce. The uncertainty, changing rules, excessive demands, prolonged working hours/days, and challenges encountered by health workers during the pandemic took a significant toll on nurses. This has resulted in increased stress, burnout, mental illness, absenteeism, and attrition among nurses [40,38].

The lessons from COVID-19 pandemic emphasise the urgency of strengthening domestic workforce resilience through improved retention, expanded training capacity, and more sustainable staffing models. Without these reforms, Ireland risks persistent dependence on migrant nurses and a continued vulnerability to external shocks.

## 5 Policy implications and directions for future research

The findings from this study demonstrate that Ireland’s continued reliance on migrant nurses is unlikely to be sustainable in the longer term. The following policy recommendations outline priority areas for action to support a more resilient, self-sufficient nursing workforce.

### 5.1 Increasing domestic nursing education capacity

A significant expansion in nursing and midwifery student places is required to address long-term supply gaps. Investment should focus on expanding Nursing School and faculty capacity, increasing clinical placement availability, and enhancing simulation-based training. National initiatives to attract applicants, including financial incentives, targeted outreach, and flexible entry pathways should be considered to further support growth in the education pipeline.

### 5.2 Integrate nursing workforce strategies with Sláintecare implementation

Workforce planning should be grounded in integrated modelling that accounts for demographic change, chronic disease trends, and Sláintecare reforms. Establishing a centralised national health workforce intelligence function would facilitate more accurate forecasting and allow policymakers to adjust training and recruitment targets in real time.

As Ireland shifts toward community-based and integrated care, targeted investment in advanced practice roles, specialist education, and community placements will be essential. Workforce policy should be fully aligned with Sláintecare goals to ensure that the nursing supply meets future service delivery requirements.

### 5.3 Improve efficiency and transparency in NMBI registration pathways

The Adaptation Programme and Aptitude Test remain the main routes for migrant nurses entering the Irish nursing workforce. Streamlining the assessment timelines, reducing administrative delays, and ensuring adequate placement capacity would enhance system responsiveness while maintaining professional standards.

Future research should build on these findings by undertaking a more detailed examination of the underlying drivers of Ireland’s sustained reliance on migrant nurses. Longitudinal studies exploring the push–pull dynamics shaping nurse migration to and from Ireland would deepen understanding of how global labour market shifts influence domestic workforce stability.

Qualitative research capturing the experiences of migrant nurses, particularly regarding their professional integration, retention, and career progression, would provide valuable insight into the factors that support or hinder long-term workforce participation. In addition, comparative analyses with countries that have successfully reduced reliance on migrant nurses could identify effective strategies for strengthening Ireland. nurse workforce (either by increasing domestic training capacity and/or improving retention).

Workforce simulation and modelling studies are also needed to project future supply and demand under alternative policy scenarios, including increased training places, improved retention measures, and reduced reliance on international recruitment. Finally, research examining the ethical and health system implications of cross-border recruitment, especially for source countries experiencing their own nurse workforce shortages, would contribute to more balanced and sustainable workforce planning more closely aligned with the WHO Global Code.

## 6 Limitations

This study has several limitations that should be considered when interpreting the findings. First, the analysis relies primarily on Nursing and Midwifery Board of Ireland (NMBI) registration data, which indicate the number of nurses entering the register but do not capture how many subsequently take up employment in Ireland or remain in the workforce over time. Registration figures therefore cannot fully reflect actual workforce participation, retention, or attrition patterns. Secondly, the study does not include qualitative data, which limits understanding of the lived experiences of migrant nurses and the reasons underpinning migration, retention, or departure. Thirdly, the analysis is descriptive and does not employ predictive modelling approaches that could estimate future supply and demand under different policy scenarios. Additionally, the study focuses on national-level trends and does not examine regional variations, sector-specific staffing differences, or disparities across clinical settings. Finally, the reliance on secondary sources may introduce inconsistencies related to variation in reporting practices across datasets and years. Despite these limitations, the study provides a valuable overview of long-term trends in nurse migration and domestic training capacity in Ireland and offers important insights for workforce planning.

## 7 Conclusion

This study provides a timely and policy-relevant assessment of Ireland’s sustained reliance on migrant nurses within the broader context of persistent national nursing shortages. By analysing trends in the demand and supply of migrant nurses between 2003 and 2022, and drawing on a diverse range of secondary data sources, the study offers a comprehensive picture of the demographic, economic, and workforce forces shaping Ireland’s nursing labour market. The integration of historical migration patterns with contemporary recruitment dynamics deepens understanding of the systemic drivers of mobility and their implications for the Irish health system.

The paper also makes an important contribution to ongoing workforce policy debates by highlighting the regulatory pathways through which migrant nurses enter practice, an aspect often overlooked in the discussions of recruitment and integration. Findings indicate that Ireland continues to depend heavily on international recruitment to maintain workforce capacity, underscoring the need for a strategic shift toward strengthening domestic training pipelines and improving retention.

Developing a robust, population-informed workforce planning system, supported by high-quality qualitative and quantitative data is essential for anticipating future healthcare needs and reducing vulnerability to global labour market fluctuations. A strong and sustainable nursing workforce is critical not only for delivering high-quality care but also for supporting the broader social and economic well-being of the country.

Overall, the study highlights the urgent need for coherent, long-term workforce strategies that balance domestic supply expansion, ethical international recruitment, and improved working conditions. Proactive and evidence-informed workforce planning will be central to ensuring that Ireland’s health system remains resilient, equitable, and capable of meeting the evolving needs of its population into the future.

## CRediT authorship contribution statement

**Comfort O. Chima:** . **Vishnu Renjith:** Writing – review & editing, Supervision, Methodology, Formal analysis, Conceptualization. **Niamh Humphries:** Writing – review & editing, Supervision, Formal analysis, Conceptualization.

## Funding

This research did not receive any specific grant from funding agencies in the public, commercial, or not-for-profit sectors.

## Declaration of competing interest

The authors declare that they have no known competing financial interests or personal relationships that could have appeared to influence the work reported in this paper.
